# Association between the Psychological Effects of Viewing Forest Landscapes and Trait Anxiety Level

**DOI:** 10.3390/ijerph17155479

**Published:** 2020-07-29

**Authors:** Chorong Song, Harumi Ikei, Bum-Jin Park, Juyoung Lee, Takahide Kagawa, Yoshifumi Miyazaki

**Affiliations:** 1 Department of Forest Resources, Kongju National University, 54 Daehak-ro, Yesan-eup, Yesan-gun, Chungcheongnam-do 32439, Korea; crsong@kongju.ac.kr; 2 Center for Environment, Health and Field Sciences, Chiba University, 6-2-1 Kashiwa-no-ha, Kashiwa, Chiba 277-0882, Japan; hikei@chiba-u.jp; 3 Department of Environment and Forest Resources, Chungnam National University, 99 Daehak-ro, Yuseong-gu, Daejeon 34134, Korea; bjpark@cnu.ac.kr; 4 Department of Landscape Architecture, Hankyong National University, 327 Jungang-ro, Anseong-si, Gyeonggi-do 17579, Korea; lohawi@gmail.com; 5 Forestry and Forest Products Research Institute, 1 Matsunosato, Tsukuba, Ibaraki 305-8687, Japan; kagawa@ffpri.affrc.go.jp

**Keywords:** psychological relaxation, trait anxiety, health-promoting environments, Profile of Mood States, preventive medicine, forest therapy

## Abstract

The aim of this study was to validate the psychological advantages of viewing forest landscapes. Moreover, the associations between trait anxiety levels and psychological responses were evaluated. A total of 650 university male students (age, 21.7 ± 1.6 years) viewed a scenery in a forested area and an urban area for 15 min. Furthermore, the Profile of Mood States questionnaire and State-Trait Anxiety Inventory were employed for the assessment of the psychological responses and the level of trait anxiety, respectively, of the participants. Results showed that compared with viewing a city area, viewing forest areas increased positive mood state, such as vigor, and decreased negative mood states. Furthermore, trait anxiety level and changes in the psychological responses such as depression–dejection, fatigue, and confusion after viewing forest landscapes were significantly correlated. The participants with high anxiety levels had greater reduction in negative mood state, including confusion, than those with low anxiety levels. In conclusion, viewing forest landscapes induced psychological relaxation, which was more evident in individuals with high anxiety levels.

## 1. Introduction

We live in an urbanized environment. Urbanization is a major factor for the improvement of living conditions [1]. However, these extremely drastic environmental changes, including increase in traffic and various pollution such as air and water and decrease in green space, threaten human well-being and health and affect quality of life [2,3]. Previous research reported that there is an association between urban living and an increased risk of health problems [4,5]. In particular, there is an increase in profound mental health problems. The prevalence rates of psychiatric disorders [6] and specific pooled rates for mood and anxiety disorders [7] were significantly higher in individuals living in urban areas than in those living in rural areas. Furthermore, city dwellers exhibit a higher risk of mood and anxiety disorders [7], and higher rates of psychotropic medication prescriptions are observed in city dwellers than in non-city dwellers [8].

Although urban living causes these health problems, the number of urban residents can still gradually increase. According to a report by the United Nations, it is expected that approximately 70% of humans will live in urban areas by 2050 [9]. It is believed that planning and creating a more comfortable urban environment in which we live will be an important issue for human well-being. The expansion of urban green space, which helps improve the well-being of humans using two approaches, can be a promising solution. The extension of nature can contribute to regulating ecosystem services, thereby preventing illness caused by harmful environmental conditions and heat and air pollution [10]. Moreover, access to nature can promote human health through physical activity and social interactions [11,12].

Recently, several studies have shown that a positive relationship exists between access to nature and human physiological and psychological well-being among urban dwellers. Demographic studies have reported that urban green spaces are positively associated with residents’ perceived general health [11,12,13,14,15]. If there is green space within walking distance of the residence, it can lead to an increase in the longevity of senior citizens [13]. As a more direct effect, contact with nature promotes psychological restoration [16] and reduces stress [17,18]. Moreover, brief walking in an urban green space, as opposed to that in a city area, induces psychological as well as physiological relaxation, as evidenced by suppressed sympathetic nervous system activity, improved parasympathetic nervous system activity, improved mood state, reduced anxiety levels, and decreased heart rate [19,20,21].

However, these studies included a small sample size, and differences in sample size have varying effects. Studies about this are extremely limited. An individualized approach is vital for creating green spaces that fit each individual’s characteristics, needs, and preferences. Conducting studies on this issue can be challenging because a large sample size is needed. Previously, we assessed individual differences in mood state changes after walking and viewing forested areas in a large cohort, and significant correlations were found between them and initial values [22]. Spending time in the forest area resulted in a reduction in negative mood states, which was more evident in individuals with high negative mood states than in those with low negative mood states.

Anxiety is a social problem in modern society. Thus, the current study aimed to validate the mental advantages of viewing real forest landscapes in a large cohort. Furthermore, the associations between changes in mood state after viewing forest landscapes and trait anxiety levels were assessed.

## 2. Materials and Methods

### 2.1. Experimental Sites and Study Period

We conducted this study from 2005 to 2013 in 56 city and forest areas in Japan. Moreover, experiments were conducted in forest areas that were safe and well-maintained. The city landscapes were situated in downtown areas. We conducted all experiments during the summer from July to September.

### 2.2. Participants

In total, 12 male Japanese university students who were recruited via a bulletin board advertisement at a university participated in each experiment (n = 672; 12 participants × 56 areas). The cohort comprised students with various majors. Thus, there was no related direct bias in the experiments. A history of physical or psychiatric disorder was not reported by any participant. The results of 22 participants were excluded due to their absence for personal reasons. Thus, the data of 650 participants aged 21.7 ± 1.6 years were analyzed.

This study protocol was approved by the institutional ethics committee of the Forestry and Forest Products Research Institute (project identification code number: 16-558; 2005–2006; 24 areas including 288 participants) and the ethics committee of the Center for Environment, Health and Field Sciences, Chiba University (project identification code number: 5; 2007–2013; 32 areas including 384 participants) in Japan.

### 2.3. Experimental Design

On the morning (13 areas) or the day prior to (43 areas) the experiment, 12 participants visited the meeting room for their orientation in each experimental region. Before the initiation of the experiments, the experimental procedures and objectives of the study were explained and written informed consent was obtained. For eliminating order effects, we randomly divided the participants into 2 groups (n = 6 for each group). On day 1, one group conducted the experiment in the forested area, whereas the other conducted the same experiment in the city area. On day 2, the groups switched the experimental sites.

**Figure 1:**
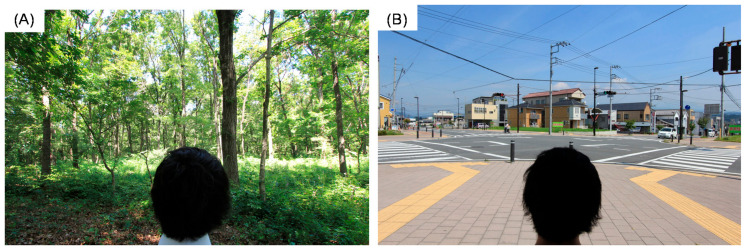
Experimental landscapes: (**A**) forested and (**B**) city areas.

Upon arrival in a city or forest areas, the participants waited for their turn inside a room and each of them was eventually taken to the experimental site. They stayed in two environments and viewed the landscape for 15 min while resting in a chair (Figure 1). After the viewing, they underwent an assessment of the Profile of Mood State (POMS) questionnaire and returned to the waiting room. Finally, a questionnaire survey was conducted to investigate the trait anxiety level of the participants.

### 2.4. Psychological Measurements

The Profile of Mood State (POMS) questionnaire is a analytically derived measure of mood state. This tool has high reliability and validity levels based on previous studies [23,24]. Moreover, the POMS can simultaneously evaluate six moods, namely tension and anxiety (T-A), depression and dejection (D), anger and hostility (A-H), fatigue (F), confusion (C), and vigor (V). A short form including 30 questions as well as the Japanese version [25] was used for reducing the burden on the participants. Furthermore, T-scores of the POMS were used for analysis.

Additionally, the State-Trait Anxiety Inventory (STAI), a self-reported tool, is used to measure the severity and presence of current anxiety symptoms [26]. Trait anxiety, involving 20 questions, measures the relatively stable anxiety proneness factors [27]. In this study, form X as well as the Japanese version was used for assessing the trait anxiety level of the participants and individuals with scores ≥44, between ≤43 and ≥33, and ≤32 were classified under the high, normal, and low anxiety groups, respectively.

### 2.5. Data Analysis

Psychological responses after viewing forest and city landscapes were compared using the Wilcoxon signed-rank test. The correlation between the POMS subscale scores after viewing forest landscapes (values after observing a forested area compared with those after observing through a city landscape) and the trait anxiety scores of STAI were evaluated using Pearson’s correlation test. Participants with high and low anxiety levels were assessed using the Mann–Whitney U test.

The Statistical Package for the Social Sciences (SPSS version 20.0, SPSS Inc., Chicago, IL, USA) was used for statistical analyses. A *p*-value of <0.05 was considered statistically significant.

## 3. Results

**Figure 2:**
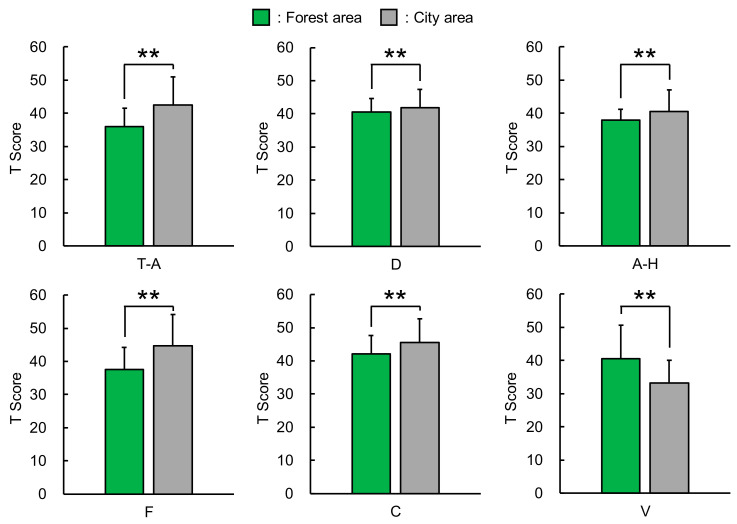
Profile of Mood States scores after observing city and forest areas. T–A, tension–anxiety; D, depression–dejection; A–H, anger–hostility; F, fatigue; C, confusion; and V, vigor. n = 649–650; mean ± standard deviation; ** *p* < 0.01 decided by the Wilcoxon signed-rank test.

Significant differences were observed in T–A, D, A–H, F, C, and V between the participants who viewed forested areas and those who viewed city areas (Figure 2). The T-A subscale score was significantly lower after observing forest areas than after observing city landscapes (mean ± standard deviation, 36.1 ± 5.5 vs. 42.6 ± 8.4; *p* < 0.01). The results for the D (forest area, 40.6 ± 4.0; city area, 41.9 ± 5.6; *p* < 0.01), A-H (forest area, 38.0 ± 3.3; city area, 40.6 ± 6.5; *p* < 0.01), F (forest area, 37.6 ± 6.7; city area, 44.7 ± 9.4; *p* < 0.01), and C (forest area, 42.1 ± 5.5; city area, 45.5 ± 7.3; *p* < 0.01) subscales were similar. The negative mood state was significantly lower after observing forested areas than after observing city landscapes. Conversely, the mean score for V after viewing forest areas was significantly higher than that after viewing city areas (40.5 ± 10.2 vs. 33.3 ± 6.9; *p* < 0.01); thus, after viewing forest landscapes, the positive mood state was observed to be higher.

Based on the assessment using STAI, of the 650 participants, 351, 247, and 52 were classified under the high, normal, low anxiety groups, respectively.

**Figure 3:**
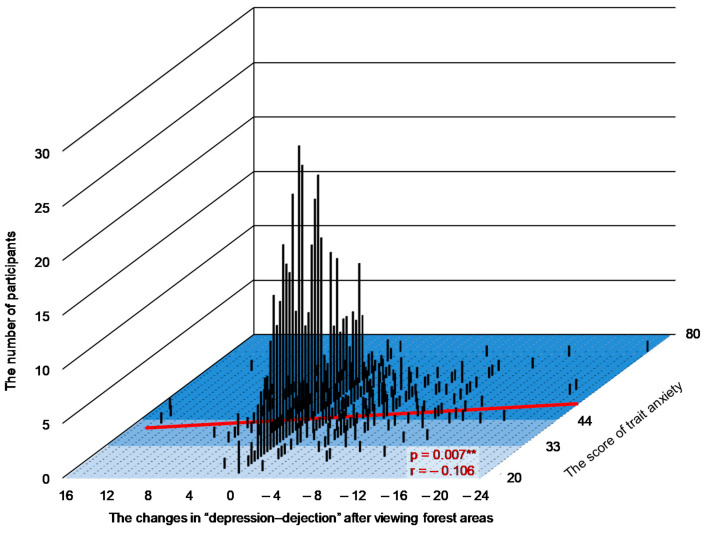
A graph indicating the relationship between changes in depression–dejection after observing forest landscapes, number of participants, and trait anxiety scores. n = 650, **: *p* < 0.01 decided by the Pearson’s correlation test.

**Figure 4:**
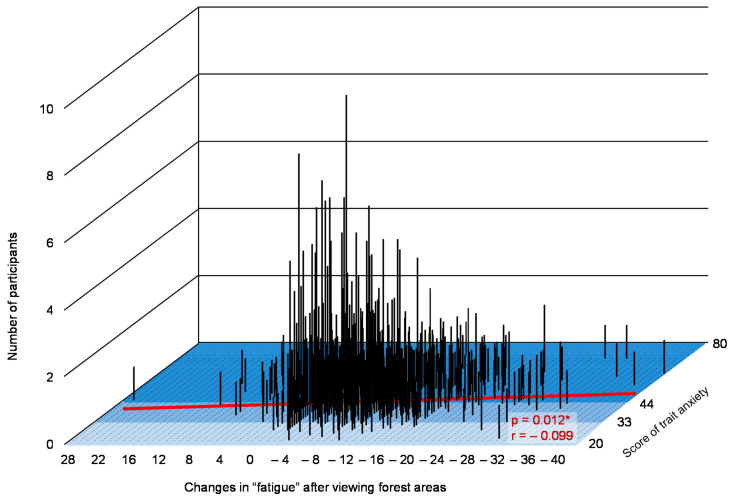
A graph indicating the relationship between changes in fatigue after observing forest landscapes, number of participants, and trait anxiety scores. n = 649, *: *p* < 0.05 decided by the Pearson’s correlation test.

**Figure 5:**
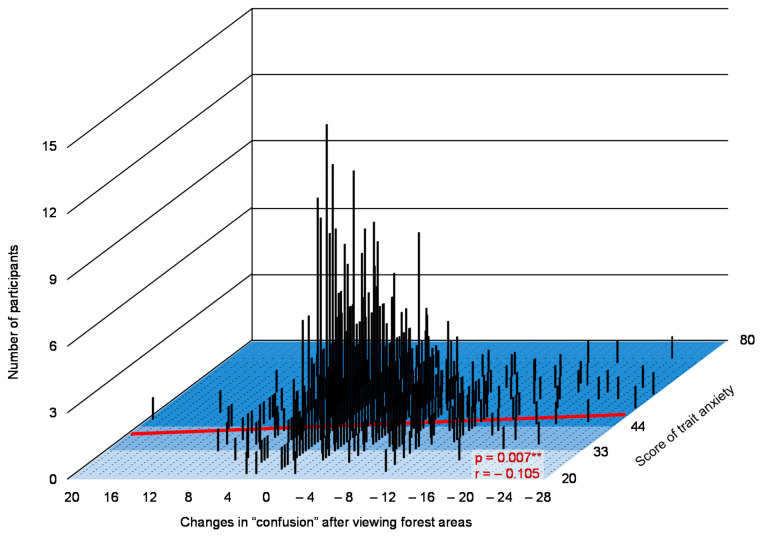
A graph indicating the relationship between changes in confusion after observing forest landscapes, number of participants, and trait anxiety scores. n = 649, **: *p* < 0.01 decided by the Pearson’s correlation test.

Changes in the D, F, C subscales after viewing forest areas (values after viewing a forest area compared with those after viewing a city landscape) and trait anxiety levels (*p* < 0.05) were significantly correlated. In the graph, the x-axis indicates changes in the D, F, C after viewing forests, y-axis indicates scores of trait anxiety, and z-axis represents the number of participants, as shown in Figure 3, Figure 4 and Figure 5, respectively.

**Figure 6:**
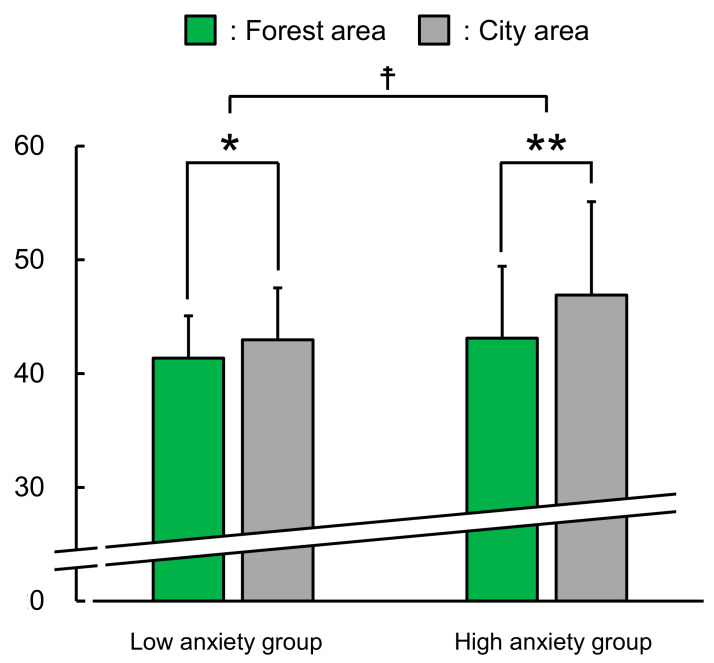
Comparison between groups with high and low anxiety levels with regard to experiencing confusion after viewing forest and city areas. Participants with low anxiety levels: n = 52; participants with high anxiety levels: n = 350. Mean ± standard deviation. ☨ *p* < 0.05 (comparing groups) decided by Mann–Whitney U test, ** *p* < 0.01, * *p* < 0.05 (comparing the two areas) decided by Wilcoxon signed-rank test.

In addition, a higher reduction in the C subscale score after viewing forest landscapes was shown in the group with high trait anxiety than in the group with low trait anxiety (*p* < 0.05, Figure 6).

## 4. Discussion

We found that viewing forest landscapes, as opposed to viewing city areas, decreased negative mood states and improved positive mood states. These results are partially coherent with previous studies on the effects of observing forest scenery or walking through forested areas [28,29,30]. We first assessed the use of a large sample size of 650, and the psychological advantages of viewing forest landscapes in this large cohort were evident.

These findings support the notion that simple access to nature for a short period may promote mental relaxation. Mental health problems are becoming more serious these days, making them a social issue. The psychological benefits of nature are extremely significant, and in the future, urban green spaces can play essential roles in promoting mental health. Nature therapy aims at achieving the effects of preventive medicine through access to nature, which renders a state of physical and mental relaxation and boosts weakened immune functions, which have been reduced due to stress in modern society, for preventing the development of some diseases [31,32,33]. Thus, it is important to establish methods that can be used promote relaxation in daily life using urban green space. The current study’s results are valuable for achieving this goal. Moreover, health policies using nature should be considered. In addition, urban planners need to pay more attention to the maintenance and increase in accessible green spaces in urban areas. Nature’s beneficial effects provide a cost-effective, simple, and accessible method for improving the quality of life and health of individuals in urban areas.

Furthermore, we found that trait anxiety levels and changes in the D, F, and C subscales of POMS after viewing forest landscapes were significantly correlated. Our results suggested that psychological responses differ based on the trait anxiety levels of a participant and a greater reduction in C after viewing forest landscapes was observed in the participants with high anxiety levels than those with low anxiety levels. These results are partly consistent with those of our previous study on the relationship between the psychological effects by a brief forest walk and trait anxiety levels [34]. In a previous study, individuals’ trait anxiety levels and changes in the D subscale of POMS by forest walk were significantly correlated. When walking through the forest, a significant correlation was found in D alone. A greater reduction in the D by forest walk was observed in participants having high trait anxiety levels than in those having normal and low trait anxiety levels. This result indicates that the psychological advantages of a brief forest walk among individuals with high trait anxiety levels can differ based on the way of enjoying the forest, such as the dynamic way of walking and the static way of viewing the scenery; this point must be considered in future studies.

The number of studies that have assessed individual differences in psychological responses is extremely low. It is particularly important in modern society to use an individualized approach for creating green spaces that fit each individual’s characteristics, needs, and preferences. More studies in this area need to be conducted. This study investigated individual differences, with a focus on the individual characteristics of “anxiety” in a large sample size. These results can be used in the future as important basic research on individual differences.

The current study had several limitations. First, for validating the psychological effects of viewing forest areas, the study was performed in representative forests of each region. Because the experiments were performed at 52 different sites, the results might have been affected by the differences according to a region. Effects based on the different characteristics of the forests, such as amount, type, and location, must be investigated in the future. Second, for generalizing the results, studies including groups with different demographic characteristics, for example sex and age, should be conducted. Moreover, such effects must be examined in populations that experience heightened states of stress in daily life. Third, for assessing the psychological effects of viewing forests, additional psychological measurements should be used. Finally, the prior expectations and experiences of participants regarding viewing forests might have influenced the results. Thus, in future research, these limitations need to be considered.

## 5. Conclusions

Viewing forest landscapes induced psychological relaxation, which was more evident in individuals having high trait anxiety levels.
